# Caseous necrosis of the mitral annulus masquerading as an intracardiac mass

**DOI:** 10.21542/gcsp.2022.1

**Published:** 2022-06-30

**Authors:** Benjamin L. Shou, Meghan E. Halub, Alice L. Zhou, Jennifer S. Lawton

**Affiliations:** Division of Cardiac Surgery, Department of Surgery, Johns Hopkins University School of Medicine, Baltimore, Maryland, USA

## Abstract

Caseous necrosis of the mitral annulus is a rare condition which typically involves the posterior leaflet or annulus. We report the case of a 71-year-old female with extensive comorbidities, presenting with dyspnea and angina, who had an incidental echocardiographic finding of a mass on or near the mitral valve. The mass increased in size over the course of three years and was associated with severe mitral regurgitation. The mass was initially thought to be a myxoma, however, surgical exploration revealed caseous necrosis of the posterior mitral annulus. Following removal of the necrotic tissue and distorted mitral leaflet, a bioprosthetic mitral valve was implanted and the patient recovered uneventfully.

## Introduction

Caseous necrosis of the mitral annulus (CNMA) is a rare condition with an estimated prevalence of 0.07% in the general population^1^. It is characterized by calcification and subsequent liquefaction of the posterior annulus or leaflet of the mitral valve^2^. The pathophysiology of CNMA is thought to first involve typical mitral annular calcification (MAC), although the pathophysiology has yet to be determined^3^. Due to its rarity and typically asymptomatic presentation, CNMA may be underdiagnosed and is often an incidental echocardiographic finding^4^. We present the case of a patient thought to have an intracardiac mass who was found to have CNMA during mitral valve replacement.

## Case Description

A 71-year-old female initially presented to the emergency room with complaints of exertional dyspnea and angina. Her past medical history was significant for stage 3 chronic kidney disease (CKD), uncontrolled hypertension (HTN), peptic ulcer disease complicated by gastrointestinal bleeding, morbid obesity (BMI of 37 kg/m^2^), hypertrophic cardiomyopathy (HCM) status post ethanol septal ablation with no residual gradient across the left ventricular outflow track, heart failure with preserved ejection fraction, mixed restrictive and obstructive pulmonary disease, nonsignificant coronary artery disease (CAD) by catheterization 6 years prior, and a previous ST-elevation myocardial infarction.

Myocardial perfusion positron emission tomography was positive for mild ischemia, and, at that time, her angina was attributed to microvascular disease. Transthoracic echocardiography (TTE) revealed diffuse sclerosis, systolic anterior motion (SAM), and trace regurgitation of the mitral valve (MV), and no masses or calcifications were noted. The left ventricle was hyperdynamic with an ejection fraction greater than 70%. She was discharged after stabilization.

Over the next 4 years, she was admitted six times, four for unstable angina or dyspnea and two for osteoarthritis. Throughout this time, the patient was nonadherent to HTN medication which frequently resulted in systolic pressures above 200 mmHg. Between 2015 and 2019, serial TTE exams demonstrated progression from mild mitral annular calcification (MAC), mild mitral regurgitation and SAM, and elevated left atrial filling pressure to moderate to severe mitral regurgitation with an eccentric jet, thickened leaflets and chordae, and a severely dilated left atrium.

Ultimately, she was admitted in January 2021 with severe exertional dyspnea and angina consistent with NYHA Class IIIb-IV heart failure symptoms. She was hypertensive with a systolic pressure 170-190 mmHg, in sinus rhythm with bifascicular block, had a mildly elevated troponin, proBNP of 1960 pg/mL, and creatinine of 2.5 mg/dL from a baseline of 2.1.

**Figure 1.**
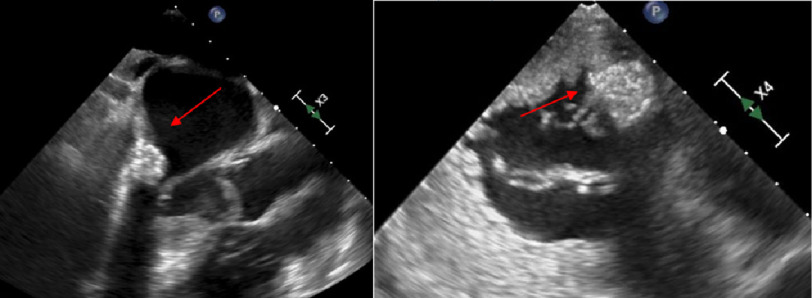
Transesophageal echocardiography imaging (left: mid-esophageal long-axis view, right: transgastric basal short axis view). Red arrows point to the circular echogenic mass, measuring 1.5 by 1.4 cm, near or on the posterior annulus of the mitral valve. There was prolapse of the P3 posterior mitral valve leaflet and severe mitral regurgitation with eccentric jets anteriorly and centrally.

Myocardial perfusion single positron emission computed tomography ruled out ischemia as the source of her symptoms. Transesophageal echocardiography (TEE) revealed a large, well-circumscribed echodense mass measuring 1.5 by 1.4 cm near the posterior mitral annulus and leaflet (Figure 1). There was prolapse of the posterior mitral valve leaflet with severe eccentric regurgitation towards the left atrial appendage, a normal anterior leaflet, and no mitral stenosis. Cardiac catheterization demonstrated no significant CAD. Her case was discussed at a multidisciplinary case conference and the differential diagnosis included myxoma, atypical MAC, calcified amorphous tumor, papillary fibroelastoma, or endocarditis. Given the severity of the mitral regurgitation and the patient’s progressively worsening functional status, mitral valve replacement was determined to be the most appropriate treatment option.

**Figure 2.**
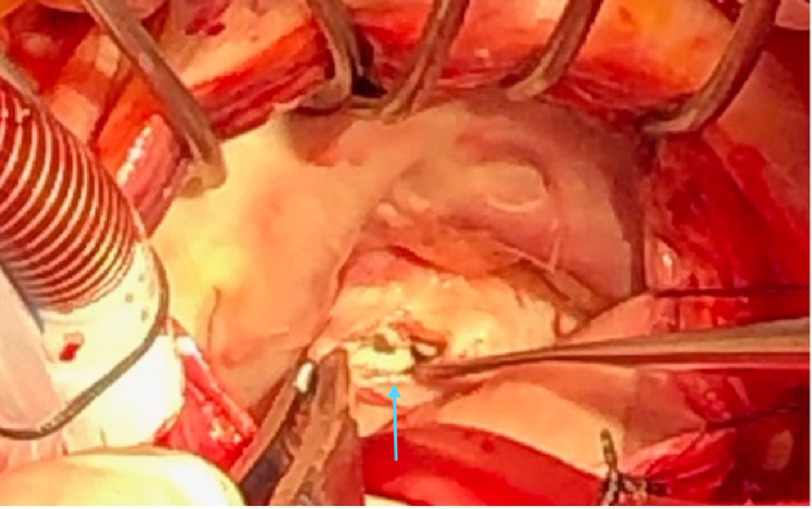
Intra-operative image of the caseous necrosis observed on the posterior leaflet of the mitral valve. A surgeon’s view of the mitral valve (patient’s head to the left and feet to the right) with exposure to the mitral valve via Waterston’s groove (left atrium). The posterior annulus was filled with caseous material (blue arrow).

During surgery, the mitral valve was approached via Waterston’s groove. The anterior mitral leaflet was significantly thickened, and the posterior leaflet had rupture of all the chordae tendinae of the P3 segment. An extensive search of the left atrium and septum was performed, and no obvious mass was found. There was a circular hard calcified area on the posterior annulus that was unroofed and found to be old caseous calcified tissue which was liquified and associated with a small cavity (Figure 2). Cultures and tissue were sent to pathology. The anterior and posterior chordae were left intact.

The remnant cavity was then completely obliterated with interrupted pledgeted 2.0 Ethibond sutures that were utilized to reconstruct the mitral annulus and utilized for placement of the mitral valve. A 27 mm pericardial valve was placed. Post operatively, the patient remained in complete heart block and had a pacemaker placed. Postoperative TTE found a well-seated, normally functioning bioprosthetic valve in the mitral position with no regurgitation. Pathology demonstrated myxoid degeneration and dystrophic calcification of the posterior leaflet. The patient was discharged on postoperative day 22.

## What have we learned?

This case highlights a patient with a complex cardiovascular history with significant comorbidities and progressively deteriorating mitral regurgitation secondary to caseous necrosis of the mitral annulus (CNMA) and ruptured chordae tendinae. Although mitral annular calcification (MAC) is estimated to occur in 5–15% of the general population^5,6^, progression to caseous necrosis of the annulus and leaflet(s) is rare and seen in only 0.6−2.7% of MAC cases^2,7^. The precise etiology of CNMA is unknown and may be related to improper calcium metabolism, hemodynamic stress, CKD, or altered bone and mineral metabolism^3,8^. It is most frequently seen in elderly women^9^ and involves the posterior mitral annulus^10^. The patient presented with mitral valve morphology, CKD, HTN, and osteoarthritis which are consistent with these potential mechanisms.

CNMA is usually asymptomatic and often misdiagnosed as a cardiac tumor during echocardiographic or computed tomography imaging^10^. However, in our case, the patient presented had progressively worsening exertional dyspnea and angina that were primarily attributed to her HCM and uncontrolled HTN prior to the echocardiographic visualization of the mass. Serial TTE over two years revealed mitral valve and annular abnormalities which were confirmed by TEE. At the time of surgery, the echodense mass was suspected to be a myxoma. Cardiac magnetic resonance imaging may have provided more insight into the lesion; however, this was not pursued due to the patient’s CKD.

The clinical course of CNMA can vary substantially. It is usually benign, but may also embolize necrotic tissue, or spontaneously resolve back to typical MAC^1,11^. In the case presented, CNMA was associated with significant valvular dysfunction which necessitated surgical intervention. With successful reconstruction of the annulus and valve replacement, the patient had resolution of mitral valve regurgitation.
